# High Apelin Level Indicates a Poor Prognostic Factor in Muscle-Invasive Bladder Cancer

**DOI:** 10.1155/2019/4586405

**Published:** 2019-03-07

**Authors:** Long Yang, Yan-Lei Li, Xiao-Qing Li, Zheng Zhang

**Affiliations:** ^1^Department of Urology, Tianjin Medical University General Hospital, No. 154, Anshan Street, Heping District, Tianjin 300052, China; ^2^Department of Pathology, Tianjin Medical University, No. 22, Qixiangtai Road, Heping District, Tianjin 300070, China; ^3^Department of Oncology, Tianjin Medical University General Hospital, No. 154, Anshan Street, Heping District, Tianjin 300052, China; ^4^Department of Urology, Tianjin Institute of Urology, The Second Hospital of Tianjin Medical University, No. 23, Pingjiang Road, Hexi District, Tianjin 300211, China

## Abstract

**Purpose:**

To compare the expression level of apelin in muscle-invasive bladder cancer and matched paracarcinoma tissues and investigate the relationship between apelin and clinical prognosis in the patients.

**Methods:**

To assess apelin expression by using immunohistochemical method compared with bladder tumors and matched paracarcinoma tissues. Subsequently, the correlation of apelin expression with the clinicopathological features of bladder cancer patients was analyzed. Kaplan-Meier survival curves method was used to analyze apelin prognostic significance for muscle-invasive bladder cancer patients (including 404 muscle-invasive bladder cancer patients and 28 normal bladder tissues, in TCGA dataset).

**Results:**

Apelin protein level was overexpressed in bladder tumor tissues compared with paracarcinoma tissues. Furthermore, high apelin expression was associated with high tumor stage (*P* < 0.05), distant metastasis (*P* < 0.05), and vascular invasion (*P* < 0.05). Kaplan-Meier curve analyses showed that the overexpression of apelin was a potential predictor of overall survival and disease-free survival.

**Conclusion:**

Apelin was upregulated in bladder tumor tissues compared with matched adjacent noncancer tissues, especially in the high tumor stage, distant metastasis, and vascular invasion. What is more, high expression of apelin in muscle-invasive bladder cancer indicates the poor prognosis. These data suggested that apelin might be a therapeutic potential biomarker in muscle-invasive bladder cancer patients.

## 1. Introduction

Bladder cancer is a common tumor of the urinary tract among the world, and 81190 patients will be diagnosed with bladder cancer in 2018, of whom 17240 cases will die [1]. In the developed countries, bladder cancer is more frequent and is the 4th cancer in men and the 9th cancer in women [2], whose treatment cost is the highest per patient of all cancers [3]. Bladder cancer can be divided into three stages: nonmuscle invasive, muscle invasive, and metastatic. About 25% of patients with bladder cancer invaded the bladder muscle layer [4]. Radical cystectomy and intravesical chemotherapy are the standard treatments for MIBC, but the five-year mortality rate remains nearly 50-70% [5, 6]. Hence, finding novel biomarkers controlling muscle-invasive bladder cancer progression will promote the development of diagnosis and therapy for muscle-invasive bladder cancer [7–12].

Apelin is an endogenous ligand for G-protein-coupled receptor which was first isolated by Tatemoto et al. in 1998 [13]. Subsequently, apelin proved to play a vital role in tumor neoangiogenesis through a paracrine effect on the host vessels and its mRNA expression level was increased in 49 tumors [14]. It has been reported that apelin overexpression was related to poor prognosis in lung cancer [15], oral cancer [16], prostate cancer [17], and gastric cancer [18]. In addition, Berta et al. demonstrate that apelin induces lymphangiogenesis and participate in lymph node metastasis [19].

Until now, no study has analyzed apelin expression among bladder cancer and matched paracarcinoma tissues. In this study, we analyzed apelin levels in bladder cancer and matched paracarcinoma tissues. In addition, we evaluated the correlations between different expression levels of apelin and clinical findings and outcome.

## 2. Materials and Methods

### 2.1. Patients and Tissue Samples

In this retrospective study, a total of 120 muscle-invasive bladder cancer patients' samples were collected, who had undergone standard radical cystectomy in the Urology Department of Tianjin Medical University General Hospital, from the year 2013 to 2017. There was no history of neoadjuvant chemotherapy or preoperative therapy in all patients. All the protocols were confirmed according to the ethical guidelines of the 1975 Helsinki Declaration, and the study was approved by the Tianjin Medical University General Hospital, China. The clinical research informed consent was signed by each patient prior to surgery.

The clinical information from TCGA public data set (including 404 bladder cancer patients and 28 normal bladder tissues; bladder urothelial carcinoma (BLCA), TCGA, Provisional (http://www.cbioportal.org/study?id=blca_tcga#summary) was collected to analyze the progression-free survival and overall survival.

### 2.2. Immunohistochemistry and Scoring

Immunohistochemistry procedure was used to analyze apelin expression. Briefly, the tissue samples were stored in formalin, followed by paraffin embedding, and were sliced into 4-micrometer sections. The sections were baked at 75°C for 1 h and immersed in xylene for 20 minutes to dewax. Then, the sections were hydrated in gradient ethanol and heated in citrate buffer for about 20 minutes to repair antigen. The sections blocked endogenous catalase using 3% hydrogen peroxide solutions at 37°C for 15 minutes. After that, the sections were incubated with primary rabbit anti-human apelin antibody (1 : 500 dilution, Abcam, UK, ab59469) overnight at 4°C in a wet box. Consecutively, the sections were incubated with goat anti-rabbit secondary antibody for 1 hour in a wet box, then the sections were dripped with substrate solution (diaminobenzidine (DAB)) until the sections were developed and then redyed in hematoxylin, dehydrated in gradient ethanol, transparentized in xylene, and coverslipped with neutral balsam. For negative control groups, PBS was used to replace the primary antibody.

The scores of apelin expression was assessed on the stained area and the intensity of the brown staining. The stained area was measured by the percentage of positive tumor cells and was scored as follows: 0, <5% positive tumor cells; 1, 5%~50% positive tumor cells; and 2, >50% positive tumor cells. The intensity of the brown staining was scored as follows: 0, negative staining or weak staining; 1, medium staining; and 2, intense staining. The total score of the stained area and the intensity of the brown staining were used to classify the expression levels: 0~2, low level and 3~4, high level. The scores was assessed by two experienced pathologists and finally reviewed by a senior pathologist.

### 2.3. Statistical Analysis

All the statistical analysis used the SPSS version 22.0 software for Windows. The correlations between clinicopathological parameters and the apelin expression at the protein level were evaluated by using the chi-square test. The progression-free survival and overall survival were measured by using the Kaplan-Meier curve method. *P* value of <0.05 was regarded as significant.

## 3. Results

### 3.1. Expression of Apelin in Bladder Tumors and Matched Paracarcinoma Tissues

**Figure 1:**
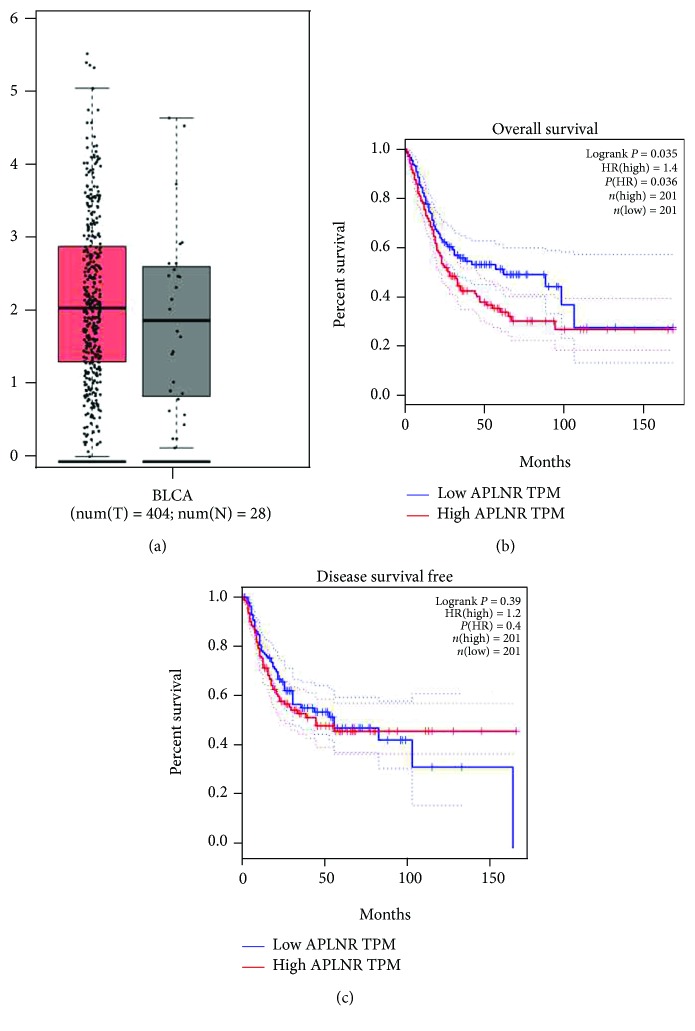
Bioinformatics analysis of apelin from the TCGA database. (a) The expression of apelin in bladder cancer tissues and normal controls. (b–c) Kaplan-Meier analysis of the associations between apelin mRNA expression and the overall survival and disease-free survival of BC patients.

The mRNA expression of apelin was evaluated by analyzing the TCGA database. As shown in Figures 1(a), there was no different expression between bladder tumor and normal bladder tissues. However, the protein expression of apelin measured by immunohistochemistry was detected in 120 bladder tumors, and matched paracarcinoma tissues showed that apelin protein was primarily located in the cell cytoplasm, which was present in bladder tumor tissues, but weakly or negatively expressed in matched paracarcinoma tissues Figures 1(a) and 1(b)).

### 3.2. Correlations between Clinicopathological Parameters and Apelin Expression

As shown in Table 1, the relationship between apelin expression and muscle-invasive bladder cancer clinicopathological features is summarized. The upregulation of apelin protein level was associated with distant metastasis (*P* < 0.05) and vascular invasion (*P* < 0.05). However, overexpression of apelin protein was not significantly associated with age (*P* > 0.05), gender (*P* > 0.05), tumor stage (*P* > 0.05), tumor grade (*P* > 0.05), lymph node metastasis (*P* > 0.05), and recurrence (*P* > 0.05).

### 3.3. Apelin Is a Poor Prognostic Factor in Bladder Cancer Patients after Radical Cystectomy

**Figure 2:**
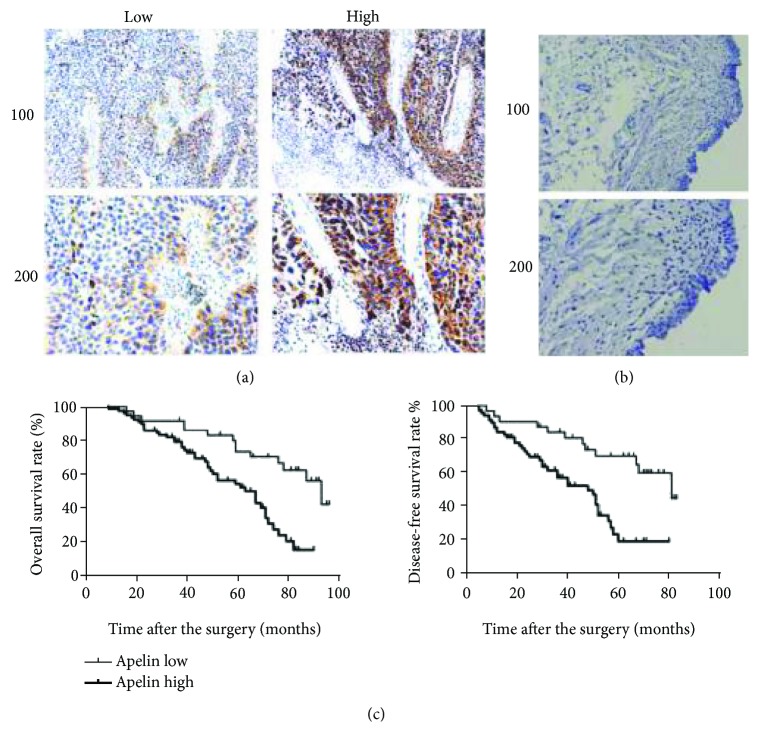
Immunohistochemical analysis of the correlation between apelin and the clinical outcomes of muscle-invasive bladder cancer patients. (a) The typical staining of high and low expressions of apelin in muscle-invasive bladder cancer tissues. (b) The negative staining of apelin in normal bladder tissues. (c) Kaplan-Meier analysis of the associations between apelin protein expression and the overall survival and disease-free survival of patients.

The data of bladder cancer patients who have undergone radical cystectomy were from TCGA dataset and Tianjin Medical University General Hospital. We used the Kaplan-Meier method to analyze the association between apelin expression and patients' survival in our hospital. Generally, we divided the patients into apelin high-expression subgroups and apelin low-expression subgroups Figure 1(a), and there was significant difference between the two groups in the patients' overall survival (*P* = 0.015 < 0.05, Figure 1(c) and disease-free survival (*P* = 0.012 < 0.05, Figure 1(c)). Consistent with our results, apelin at mRNA level was present both in bladder tumor tissues and the matched paracarcinoma tissues Figure 2(a). We also found that high apelin mRNA expression was significantly related with patients' overall survival (*P* = 0.035 < 0.05, in the TCGA dataset, Figure 2(b)) but not disease-free survival (*P* = 0.39 > 0.05, in the TCGA dataset, Figure 2(c)).

## 4. Discussions

The therapies and survival rate for muscle-invasive bladder cancer patients have improved with using ultrasonic examination [20], cystoscopy [21], and urine exfoliative cytology [22]. However, more and more evidence shows that the above methods may miss malignant tumors due to varied reasons [23]. Patients with metastatic and invasive events will lead to adverse prognosis and death [24]. To identify a novel biomarker for evaluating the phenotypes of muscle-invasive bladder cancer is urgent. As far as we know, clinical significance of apelin in muscle-invasive bladder cancer patients has not been reported.

In our study, we proved for the first time that apelin could play an important role in the progression of muscle-invasive bladder cancer. We found that apelin protein level was upregulated in muscle-invasive bladder cancer and high level expression of apelin was correlated with high tumor stage, distant metastasis, vascular invasion, and poor survival in muscle-invasive bladder cancer patients. These findings indicated that apelin might be a therapy target for muscle-invasive bladder cancer and may guide the urologist to better understand the progression of muscle-invasive bladder cancer.

Apelin was found to play an important role to promote tumor neoangiogenesis and sustain tumor expansion and progression in various human cancers. For example, Harford-Wright et al. [25] reported that apelin was a central regulator to maintain the stem-like properties of glioblastoma cells. To block the apelin receptor signaling pathway may suppress tumor cell expansion and progression. Meanwhile, our data also showed that apelin was a druggable factor in muscle-invasive bladder cancer patients through evaluating apelin expression and muscle-invasive bladder cancer clinicopathological features. Chen et al. [26] demonstrated a novel association between JAG/Notch3 and apelin system, targeting them can inhibit progression of colon carcinoma. Furthermore, they found that Apelin13 can upregulate the expression of Notch3. Inhibition in either of them would prevent the growth of tumor cells. Although we did not study the underlying mechanism that how apelin could promote the progression in bladder cancer, our data about apelin expression and clinicopathological features showed that apelin was a therapeutic and management target which was consistent with their findings. Feng et al. [18] showed that tumor apelin expression was significantly upregulated in gastric cancer compared with adjacent normal tissues. Our data also reported that tumor apelin protein level was highly expressed in bladder cancer by using immunohistochemistry, but there was no difference in mRNA level through analyzing the TCGA database. We thought that the protein level was more compelling, but our study was single-center with small sample, and we also need to expand the sample size in multiple centers for further analysis. In addition, the study reported that high apelin expression was closely associated with high invasion and metastatic phenotype of gastric cancer: they also conducted that high expression of apelin was correlated with poor prognosis and shorter overall survival in gastric patients [18]. In our study, we found that high expression of apelin was also correlated with shorter overall survival and disease-free survival in muscle-invasive bladder cancer patients. In Hall et al.'s study [27], they found that apelin was upregulated in cholangiocarcinoma in the protein level by using IHC compared to adjacent nonmalignant liver tissues. They also found apelin gene expression was increased in human cholangiocarcinoma by RT-qPCR. So they used ML221, an apelin receptor antagonist, to treat tumor-bearing mice, and observed that ML221 treatment was effective on decreasing tumor growth. Hoffmann et al. [28] found that mRNA and protein expressions of apelin and its receptor were higher in ovarian tumor cell lines compared with noncancer cell lines. Apelin was involved in mitosis in ovarian epithelial cancer cells. With these previous findings in glioblastoma, colon carcinoma, gastric cancer, cholangiocarcinoma, and ovarian cancer, our data also found that high expression of apelin was significantly associated with invasion and metastatic features and poor prognosis in patients with muscle-invasive bladder cancer.

Lastly, our data also showed that apelin was upregulated in bladder tumor tissues compared with matched adjacent noncancer tissues, especially in the high tumor stage, distant metastasis, and vascular invasion. What is more, high expression of apelin in muscle-invasive bladder cancer was correlated with the poor prognosis. These data suggested that apelin might be a potential therapeutic biomarker in muscle-invasive bladder cancer patients. But our study is single-centered with small sample; the sample size in multiple centers should be expanded for further verification. It is urgent to conduct cell experiments in vitro and animal experiments in vivo to comprehend the underlying mechanism of apelin's upstream and downstream interactions in muscle-invasive bladder cancer progression.

## Figures and Tables

**Table 1:** Relationships of apelin and clinicopathological characteristics in 120 patients with muscle-invasive bladder cancer.

Feature	All *n* = 120	Apelin expression		*χ* ^2^	*P*
		Low	High		
		*n* = 40	*n* = 80		
Age (years)				1.406	0.236
<65	67	27	45		
≥65	53	13	35		
Gender				0.677	0.410
Male	64	24	40		
Female	56	17	39		
Tumor stage				5.419	0.020^∗^
T2	40	19	21		
T3/T4	80	21	59		
Tumor grade				0.944	0.331
Low	38	15	23		
High	82	25	57		
Lymph node metastasis				0.957	0.328
Yes	37	10	27		
No	83	30	53		
Recurrence				1.678	0.195
Yes	55	15	40		
No	65	25	40		
Distant metastasis				5.625	0.018^∗^
Yes	48	10	38		
No	72	30	42		
Vascular invasion				4.344	0.037^∗^
Yes	52	12	40		
No	68	28	40		

∗ indicates *P* < 0.05 with statistical significance.

## Data Availability

The data used to support the findings of this study are available from the corresponding author upon request.
