# Corrigendum to “High Apelin Level Indicates a Poor Prognostic Factor in Muscle-Invasive Bladder Cancer”

**DOI:** 10.1155/2019/5974238

**Published:** 2019-08-28

**Authors:** Long Yang, Yan-Lei Li, Xiao-Qing Li, Zheng Zhang

**Affiliations:** ^1^Department of Urology, Tianjin Medical University General Hospital, No. 154, Anshan Street, Heping District, Tianjin 300052, China; ^2^Department of Pathology, Tianjin Medical University, No. 22, Qixiangtai Road, Heping District, Tianjin 300070, China; ^3^Department of Oncology, Tianjin Medical University General Hospital, No. 154, Anshan Street, Heping District, Tianjin 300052, China; ^4^Department of Urology, Tianjin Institute of Urology, The Second Hospital of Tianjin Medical University, No. 23, Pingjiang Road, Hexi District, Tianjin 300211, China

In the article titled “High Apelin Level Indicates a Poor Prognostic Factor in Muscle-Invasive Bladder Cancer” [1], the sentence “In this retrospective study, a total of 120 muscle-invasive bladder cancer patients' samples were collected, who had undergone standard radical cystectomy in the Urology Department of Tianjin Medical University General Hospital, from the year 2013 to 2017” should be changed to “In this retrospective study, a total of 120 muscle-invasive bladder cancer patients' samples were collected, who had undergone standard radical cystectomy in the Urology Department of Tianjin Medical University General Hospital and the Second Hospital of Tianjin Medical University, from the year 2013 to 2017.”

And the sentence “All the protocols were confirmed according to the ethical guidelines of the 1975 Helsinki Declaration, and the study was approved by the Tianjin Medical University General Hospital, China” should be changed to “All the protocols were confirmed according to the ethical guidelines of the 1975 Helsinki Declaration, and the study was approved by the Tianjin Medical University General Hospital and the Second Hospital of Tianjin Medical University, China.”

In the Results section, the sentence “The data of bladder cancer patients who have undergone radical cystectomy were from TCGA dataset and Tianjin Medical University General Hospital” should be changed to “The data of bladder cancer patients who have undergone radical cystectomy were from TCGA dataset and Tianjin Medical University General Hospital and the Second Hospital of Tianjin Medical University.”
